# Ocellar structure and neural innervation in the honeybee

**DOI:** 10.3389/fnana.2014.00006

**Published:** 2014-02-19

**Authors:** Yu-Shan Hung, Michael R. Ibbotson

**Affiliations:** ^1^National Vision Research Institute, Australian College of OptometryCarlton, VIC, Australia; ^2^Department of Optometry and Vision Sciences, Centre of Excellence for Integrative Brain Function, University of MelbourneParkville, VIC, Australia

**Keywords:** honeybees, ocelli, descending neurons, ocellar resolution, focal length

## Abstract

Honeybees have a visual system composed of three ocelli (simple eyes) located on the top of the head, in addition to two large compound eyes. Although experiments have been conducted to investigate the role of the ocelli within the visual system, their optical characteristics, and function remain controversial. In this study, we created three-dimensional (3-D) reconstructions of the honeybee ocelli, conducted optical measurements and filled ocellar descending neurons to assist in determining the role of ocelli in honeybees. In both the median and lateral ocelli, the ocellar retinas can be divided into dorsal and ventral parts. Using the 3-D model we were able to assess the viewing angles of the retinas. The dorsal retinas view the horizon while the ventral retinas view the sky, suggesting quite different roles in attitude control. We used the hanging drop technique to assess the spatial resolution of the retinas. The lateral ocelli have significantly higher spatial resolution compared to the median ocellus. In addition, we established which ocellar retinas provide the input to five pairs of large ocellar descending neurons. We found that four of the neuron pairs have their dendritic fields in the dorsal retinas of the lateral ocelli, while the fifth has fine dendrites in the ventral retina. One of the neuron pairs also sends very fine dendrites into the border region between the dorsal and ventral retinas of the median ocellus.

## Introduction

Honeybees (*Apis mellifera*), like most of the flying insects possess two visual systems: the compound eyes and a set of simple lens eyes (ocelli). The morphology of the ocelli varies across insect orders and even within the same family (for review, Goodman, 1981). The lack of optical resolving power, however, represents one morphological feature that is consistently observed in ocelli of different insects. Homann (1924) examined the ocelli in the red wood ant and hover fly and found that the focal planes of the ocellar lenses were well beyond the proximal limit of the retina (i.e., the images cannot be focused on the retina). The same feature was subsequently found in other species (e.g., locust: Parry, 1947; Cornwell, 1955; Wilson, 1978; Berry et al., 2007c; blowfly: Cornwell, 1955; Schuppe and Hengstenberg, 1993; nocturnal bee and diurnal wasp: Warrant et al., 2006). The function of these under-focused lenses is not yet entirely clear, although speculation about their role began over 100 years ago (Müller, 1826; Lowne, 1870). Wilson (1978) proposed a convincing hypothesis of ocellar function based on locust ocelli, referred to as the “single-sensor” hypothesis (Stange et al., 2002). It suggests that the ocelli do not resolve spatial details of the environment: instead, each ocellus functions as a highly sensitive light detector of illumination levels from a wide region of visual space. Their large aperture and field of view suggest that they are designed to detect overall brightness while minimizing the effect of small objects in the visual field (Wilson, 1978).

However, even though the focal planes of many ocellar lenses lie behind the retina, this does not exclude the possibility of detecting form, or moving objects. Schuppe and Hengstenberg (1993) examined blowfly ocelli (*Calliphora erythrocephala*) and found that despite the under focussing of the ocellar lens, low spatial frequency patterns could be imaged on the retinal plane. Other studies have also shown that ocelli are able to resolve some spatial information, e.g., in wasps (Warrant et al., 2006) and dragonflies (Stange et al., 2002; Berry et al., 2006, 2007b,c; van Kleef et al., 2008). The capacity for some ocelli to resolve spatial features suggests that ocellar function may be more complex than previously suggested by the single-sensor theory.

The honeybee ocelli are located as a triplet on the dorsal surface of the head between the compound eyes; each ocellus consists of approximately 800 retinal cells (Toh and Kuwabara, 1974). Although the basic morphology and anatomy of the honeybee ocelli has been studied previously (Toh and Kuwabara, 1974; Pan, 1980; Ribi et al., 2011), these investigations were focused on ocellar structure in relation to the synaptic terminals of ocellar nerve fibers. The optical characteristics of the honeybee ocelli remain unclear and controversial. Ribi et al. (2011) described the anatomical structures of the median and lateral ocelli of the honeybee, making two primary observations. First, the ocellar retinas are divided into two segments: dorsal and ventral retinas. Second, the focal planes of the lateral ocellar lenses are beyond the proximal limit of the retinas but the focal plane of the median ocellar lens was within the retinas, implying that the median ocellus is capable of relatively high spatial resolution. This latter observation was contradictory to our own observations. Therefore, in this study, we re-examined the internal morphologies of the ocellar lens and retinal structure by combining serial sections and three-dimensional reconstructions of honeybee ocelli. Additionally, the dioptrics of honeybee ocelli are investigated using optical techniques. We measured the spatial resolving capacity of the ocellar retinas and created a three-dimensional (3-D) model of the ocelli that offers the capacity to observe the full structure of the lenses and their relationship with the associated retinas. This level of analysis allows us to make predictions about the viewing angles of the ocelli and, therefore, comment on their potential roles in orientation stabilization behavior. In contradiction to Ribi et al. (2011), we show that the focal plane of the median ocellus is in fact well beyond the proximal limits of the retina and that the lateral ocelli have better spatial resolution than the median ocellus.

In addition to the structural and 3-D investigations, we also filled the five pairs of large ocellar thoracic descending neurons, known as L_*D*_ neurons (Pan and Goodman, 1977; Goodman, 1981; Milde, 1984; Milde and Homberg, 1984). We show for the first time that four of these neurons have their primary dendritic fields in the dorsal retinas of the lateral ocelli, while the fifth sends fine dendrites into the ventral retina. One of the L_*D*_ neurons also sends branches into the border regions between the dorsal and ventral retinas of the median ocellus. This analysis assists in determining the role of the ocelli in delivering rapid visual information to the thoracic motor centers.

## Methods and materials

### Experimental animals

Experiments were conducted on worker honeybees, *Apis mellifera*, that had been actively foraging. Bees were collected from hive entrances or were wild-caught in local parkland. Prior to experimental preparation, each bee was lightly anaesthetized by cooling in a refrigerator at 5°C for about 20 min.

### Histology

Light microscopy was performed using standard methods. Freshly removed heads were partially dissected to remove the mouthparts, frons and the cuticle from the back of the head. The dissected head capsule was kept in primary fixative (2.5% glutaraldehyde and 4% paraformaldehyde in 1M phosphate buffer saline, PBS) for 3 h. After fixation, the samples were post-fixed in 1% phosphate buffered osmium tetroxide to enhance the contrast, and through an ethanol series (50–100%) for dehydration. The samples were then embedded in hard Epoxy resin (Epon® 812) and carefully oriented at different angles for sectioning. Semi-thin sections of 1 μm were cut using either a glass or diamond knife on a Reichert-Jung ultramicrotome. Sections were post-stained with toluidine blue and imaged on a Zeiss Axioskop (light microscope) with a SPOT RT digital camera.

### Three-dimensional reconstructions of honeybee ocelli

A selected set of frontal serial sections (1 μm thick slices) of the honeybee head was used to generate three-dimensional (3D) reconstructions using Amira 5.3.3 (Visage Imaging GmbH). The images of each slice were manually aligned relative to each other, and then segmented into discrete components by manually tracing outlines of the cuticle, lenses and retinas. Mesh models of each structure were then generated from the segmented images.

### Focal length measurement

The hanging drop method was used to measure the optics of honeybee median and lateral ocelli (Homann, 1924). The lenses of the median and lateral ocelli were carefully dissected and cleaned using honeybee saline (111.22 mM NaCl, 3.35 mM KCl, 1.37 mM CaCl_2_, and 1.89 mM Na_2_CO_3_) to minimize osmotic flow out of or into the lens. The ocellar lenses were then suspended from a droplet of bee saline, which hung below a glass cover slip. The lens was oriented with the inner surface (i.e., the back of the lens) facing the saline, and the outer surface facing the air. The cover slip with the lens was then sealed onto a rubber O-ring attached to a glass slide with vacuum grease. The distances between the inner surfaces of the lens to the best-focused plane (Back Focal Distance, BFD) were determined using grating patterns (and compared to a blank control stimulus with mean luminance the same as grating patterns).

To investigate grating resolution, an LCD flat-screen monitor was used to deliver circular or linear wide-field sine wave modulated black and green gratings at several spatial wavelengths (1, 2, 4, 8, 12, and 20°), utilizing a 50% duty cycle, such that the average intensity of light passing through the grating was constant. As the tested pattern was displayed on the LED monitor covering a wide visual angle (83.64° in horizontal direction, and 90° in vertical direction), the grating patterns were mathematically wrapped (according to the distance between the ocellar lens and the LED display) to maintain a constant angular size from the position of the ocellar lens. The screen was placed 9.5 cm from the lens. The distance required to shift the focus of the microscope objective from the inner surface of the lens to the best focal plane was measured. The results were then corrected by multiplying by the reflective index of the immersion medium (1.34 for saline). The images formed by the ocellar lenses were captured by a digital camera at different distances from the back of the lens (Back Distances, BD).

In the present study, the contrast of the digital images was calculated to determine the quality of the images, as poor contrast causes loss of detail. Images of different spatial wavelengths through the ocellar lenses were analyzed to determine the contrast present in the image. The maximum (*I*_max_) and minimum (*I*_min_) pixel values from the image were determined and used to calculate the contrast (*m*) present in the image using the Michelson equation:

(1) m=(Imax−Imin)/(Imax+Imin)

### Neuroanatomy

The morphologies of the neurons were obtained by combining fluorescent dye-filling and optical sectioning (confocal imaging). The honeybee was placed horizontally (dorsal side up) on a metal holder, and the head and thorax were secured with a 3:1 mixture of beeswax and violin resin. To give access to the ocellar descending neurons, the ventral nerve cord was exposed from the dorsal side of the neck, between the suboesophageal and prothoracic ganglia. A glass pipette filled with Texas Red (Invitrogen™) was inserted dorsally into the ventral nerve cord. The glass pipette was lowered into the live animal at room temperature for at least 2 h to allow dye uptake and diffusion through the neurons. After the dye injection, the brain and suboesophageal ganglion were carefully dissected in fixative (3.7% paraformaldehyde in phosphate buffered saline) and then transferred to 3.7% paraformaldehyde in methanol for 45 min. The samples were then dehydrated through an ethanol series of 80, 90, and 100% for 10 min each, and mounted in methyl salicylate (Sigma-Aldrich). Filled neurons were observed and optically sectioned (1 μm thickness) on a confocal microscope (LSM Pascal, Zeiss) with a 10× objective lens (numerical aperture = 0.45; Apochromat, Carl Zeiss). We used a laser wavelength of 543 nm to elicit the dye emission and the images were collected with a pixel size of 0.82 μm. The image series were processed in Zeiss LSM image viewer and AxioVision software to produce 2D projections of the brain at different angles. The images of serial scanning (cross sections) were also projected every 5–10 μm to show the neural structures in different depths. The axons and dendrites of the ocellar descending neurons were manually traced out in the stacks of projected images in Photoshop. In this study, although many more were used to achieve these fills, ocellar descending neurons were successfully filled in five specimens.

## Results

### Ocellar lenses and retinal structure

**Figure 1:**
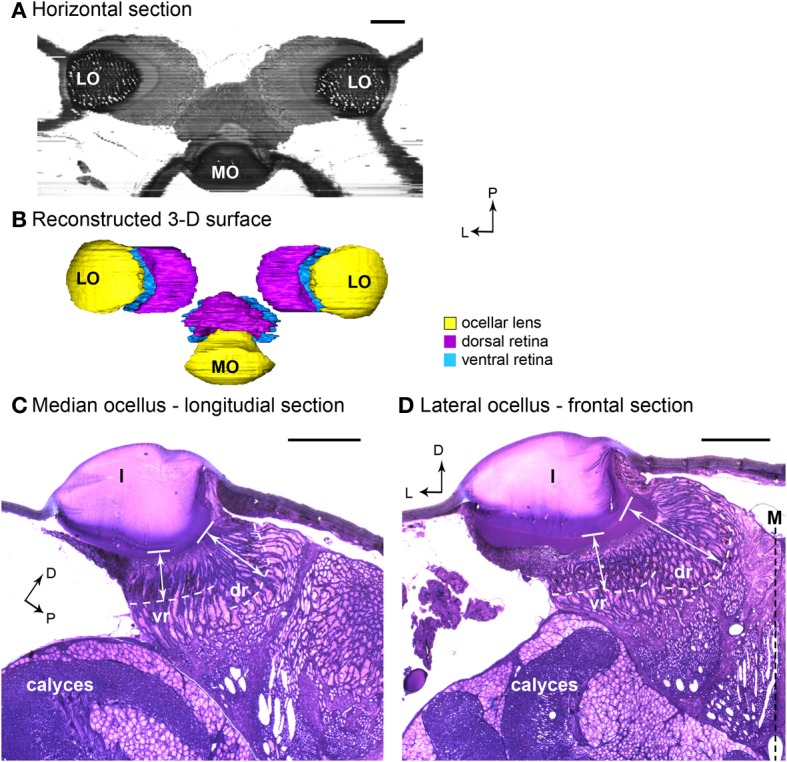
**Light micrographs of honeybee ocellar semi-thin sections. (A)** Horizontal plane of the three ocelli reconstructed from serial sections. The lens and retina of the median ocellus project dorsally while the lateral ocelli project slightly ventrally and toward the midline of the brain. **(B)** 3-D reconstructed model of the dorsal ocelli of the honeybee. **(C)** Longitudinal section of the median ocellus. The lens is a thick, dome shaped, spherical biconvex corneal lens. **(D)** Cross section of the lateral ocellus. The outer surface of the ocellar lens is smooth and convex, but the inner surface curves asymmetrically toward the midline of the brain **(**M). The white dashed lines in **(C)** and **(D)** indicate the edge of the screening pigment of the retina. Note that the dorsal orientation has been tilted to the right in **(C)** to make comparisons with **(D)** easier. In both median and lateral ocelli, the retina is divided into two regions (dr and vr). The white double-headed arrows indicate the distances from the inner surface of the lens (solid line) to the limit of the rhabodomeric zone (white dashed line). Abbreviations: D, dorsal; dr, dorsal retina; l, lens; L, lateral; LO, lateral ocellus; M, midline of the brain; MO, median ocellus; P, posterior; vr, ventral retina. Scale bars: all 100 μm.

**Figure 2:**
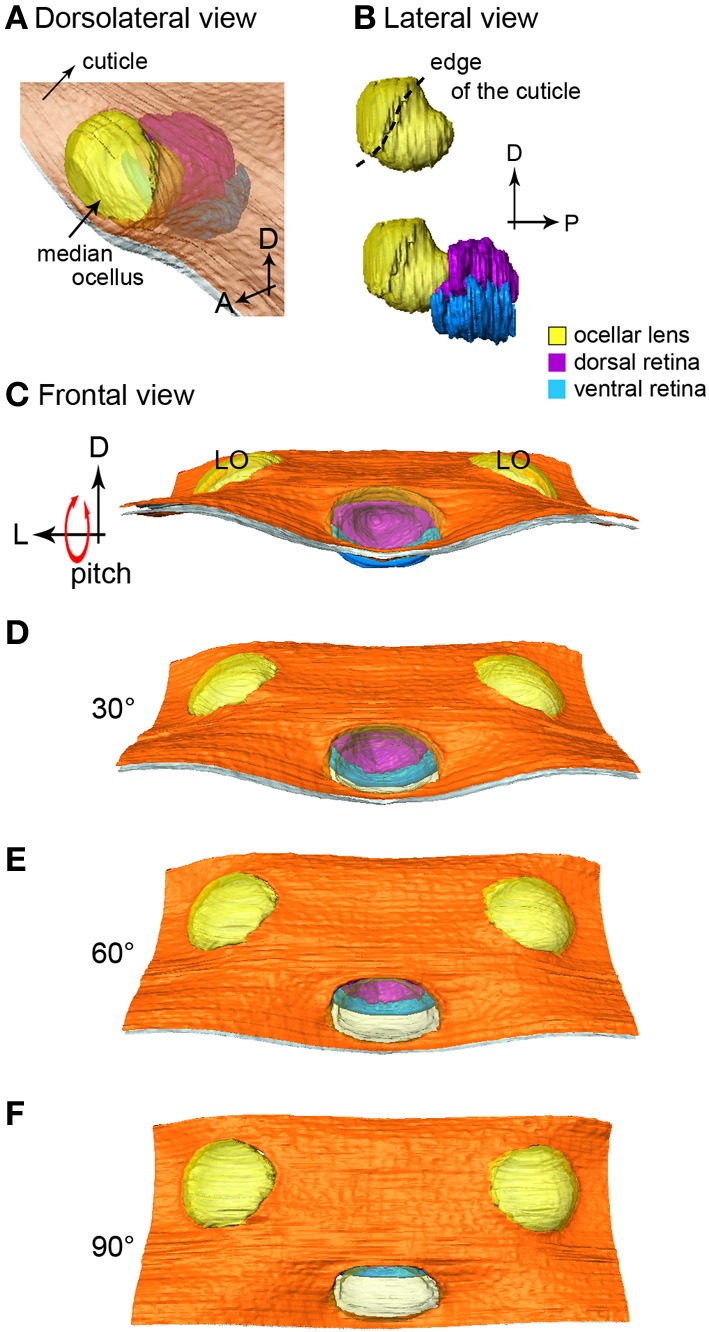
**The 3-D reconstruction of the honeybee median ocellus. (A)** Dorso-lateral view of the median ocellus with the cuticle in view. **(B)** Lateral views of the median ocellar lens (l, shown in yellow) and dorsal and ventral retinas. The lens of the median ocellus is elongated downward, the retinas are positioned downwards and posteriorly in relation to the lens. **(C–F)** Simulation of a pitching movement of the model. **(C)** A frontal view of the median ocellus: only the dorsal retina can be seen through the median ocellar lens (the ventral retina is under the cuticle). **(F)** A dorsal view of the ocelli: only the ventral retina can be seen. Abbreviations: D, dorsal; L, lateral; LO, lateral ocellus; P, posterior.

**Figure 3:**
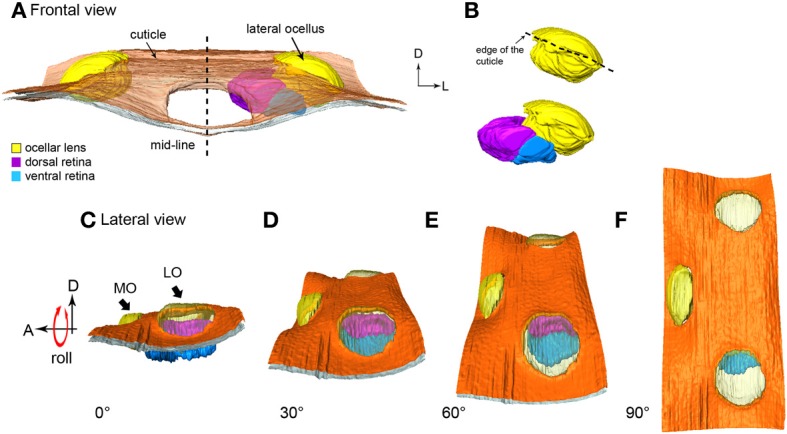
**3-D reconstruction of the honeybee lateral ocellus. (A,B)** frontal views of the lateral ocellar lens and retinas. **(A)** the inner surface of the lens (l, shown in yellow) of the lateral ocellus is asymmetrical, the dorsal (dr, shown in purple), and ventral (vr, blue) retinas follow the inner surface of the lens and are pointing toward the midline of the brain. **(C–F)** Simulation of the rolling movement of the model. The ocelli are viewed from the side of the head. To the left the ocelli are dorsal. To the extreme right, the head has been rolled downwards by 90° to reveal all three ocellar regions (only the left ocellar retinas are present). **(C)** A lateral view of the lateral ocellus: only the dorsal retina can be observed. **(F)** A dorsal view of the three ocelli: only the ventral retina can be observed. Abbreviations: A, anterior; D, dorsal; L, lateral; LO, lateral ocellus; MO, median ocellus.

From the frontal serial sections, we were able to construct a 3-D model of the honeybee ocellar lenses and retinas (Figures 1A,B). The lens and the retinas of the median ocellus project posteriorly while the lateral ocelli project slightly ventrally and toward the midline of the brain (Figures 1A,B). While sections of the three ocelli illustrate both their internal and external morphologies (Figures 1C,D), the 3-D models of the ocellar lenses and the retinal tissue that lies below the lenses also help to reveal the viewing angles of each ocellus (Figures 2, 3). Combining the results from the 3-D reconstructions and the 2-D sections, we provide a complete visualization of the retinal structures and their relation to the lenses.

In both the median and lateral ocelli of honeybee we identified two retinas, the dorsal and ventral retinas. The retinas are distinguished by the length of the retinula cells and their position relative to the lens (Figures 1C,D). We measured the distances from the inner surface of the lens to the limit of the rhabodomeric zone (marked by pigment accumulated along the edges of the retina; see Figures 1C,D) from sections of two different preparations. In Figures 1C,D, these distances are indicated by the white double-headed arrows. The measurements indicate where the retinal levels are in relation to the inner surface of the lens.

#### Median ocellus

The 3-D model reveals that below the level of the surrounding cuticle of the median ocellar lens, the inner surface of the lens curves downward and forms a complex non-spherical shape (Figures 2A,B). Measurements from two different preparations (longitudinal sections) show that the distance from the inner surface of the lens to the limit of the rhabodomeric zone is 92 μm for the dorsal retina and 55 μm for the ventral retina (*n* = 2).

In Figures 2C–F the median ocellus is seen from the front of the head (as if the bee were flying toward the observer). The simulation shows what would be seen from the frontal view as the bee makes a pitching movement (dorsal downwards and forwards). It is clear that when the head is in its normal position (Figure 2C) only the dorsal retina (purple) can be seen through the lens aperture. As the head tilts forward, more of the ventral retina becomes evident. By the time the head is tilted forward by 90° (Figure 2F), only the ventral retina (blue) is visible from the front. With the animal in its normal attitude, the dorsal retina views the horizon, while the ventral retina views the sky directly above the head. From the positioning of the lens in relation to the retinas, it can also be concluded that the retinas of the median ocellus maximize the view of the frontal visual field. A video showing the simulated roll of the head is provided in the supplementary material.

#### Lateral ocelli

From the sections it can be determined that the outer surface of the lateral ocellar lens is convex, but the inner surface of the lens curves slightly toward the midline of the brain, as it becomes more asymmetrical (Figure 1D). This feature is shown clearly by the 3-D reconstruction: under the surrounding cuticle of the lateral ocellar lens, the inner surface curves and becomes thicker toward the midline of the brain (Figures 3A,B). It is also shown that the dorsal retina (shown in purple) and ventral retina (shown in blue) follow the inner surface of the lens and their axial orientation is aimed toward the middle of the brain (Figures 3A,B). The rhabdomeric zone follows the asymmetrical inner surface, where the thickening of the lens is correlated to the thickening of the retina. Measurements from two different preparations (cross sections) show that the distance from the inner surface of the lens to the limit of the rhabodomeric zone is 148 μm for the dorsal retina and 78 μm for the ventral retina (*n* = 2).

Figures 3C–F simulates a rolling movement of the head. The observer is viewing the bee from its left side. The left-most image shows the head in the normal position (Figure 3C, ocelli uppermost). Only the purple dorsal retina is in view. Even taking refraction into account, it is unlikely that the ventral retina would be able to obtain very much information from the horizon. As the head rolls to the side (dorsal toward the viewer) the dorsal retina slowly turns out of view and the ventral retina becomes evident. When the head is turned fully through 90°, such that the top of the head points directly toward the viewer, only the ventral retina can be seen (Figure 3F). The simulation shows that when the bee head is in its normal attitude, the dorsal retina is positioned to observe the horizon while the ventral retina views directly upwards toward the sky. In other words, the dorsal retina has very little input from above the head. As the lateral ocellar retinas are positioned inwards toward the midline of the brain, it also indicates that the frontal visual fields for the lateral ocelli are very limited. A video showing simulated roll of the head is provided in the supplementary material.

### Focal length of the ocellar lenses

**Figure 4:**
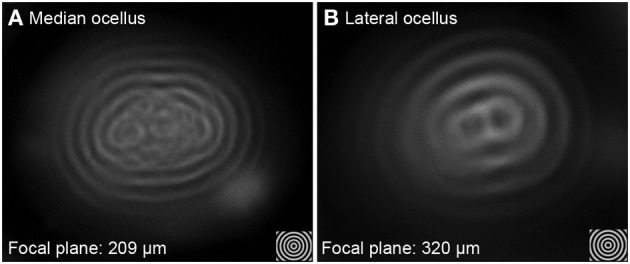
**Imaging of concentric circles through the median (A) and lateral ocellar (B) lenses of the honeybee**. Both **(A,B)** show images of angularly corrected stimuli of 5° spatial wavelength on a wide-field LCD monitor as seen through the lenses at the focal plane. Both the median and lateral ocellar lenses form a single image but the centers of the images degrade in both cases.

Utilizing the hanging-drop method to directly examine the image produced by the ocellar lenses revealed differences in resolving power (Figure 4). For example, when testing a series of concentric circles (spatial frequency = 0.2 cycles per degree, cpd) at the best focal plane, images formed by the median ocellar lens split into fragments in the middle (Figure 4A), while the lateral ocellar lens formed a better image, which shows a figure-of-eight shape in the middle (Figure 4B). The results hint that the lateral ocelli have better spatial resolution than the median ocellus.

Measuring the focal length revealed that the focal distance of the lateral ocellus is significantly longer than that of the median ocellus [*F*_(1, 65)_ = 166.73, *p* < 0.001]. The distances between the back of the lens to the focal plane (Back Focal Distance, BFD) is 209 ± 40 μm for the median ocellus and 320 ± 46 μm for the lateral ocelli (mean ± s.d., *n* = 10). As the rhabdoms are no more than 100 μm and 150 μm behind the median and lateral ocellar inner surfaces, respectively, this demonstrates that the honeybee ocellar lenses form a focal plane beyond the proximal limit of the retina in both cases.

The ocellar sections and BFD measurements show that the ocellar lenses of honeybees are under-focused with respect to the retina. The focal planes are roughly 100 μm beyond the retinal level in both the median and lateral ocelli. Therefore, imaging through the ocellar lenses at the retinal level was examined using the hanging drop method to verify the spatial resolving power of honeybee ocelli. The images formed by wide-field gratings with different spatial wavelengths were recorded at the level of the retinas with a digital camera. In all cases the resolving capacity was studied for both vertical and horizontal gratings.

To provide accurate measurements of spatial resolution over a wide range of distances from the inner surface of the lens (Back Distance, BD), photographs of the gratings were taken through the lenses at 20 μm intervals from the back of the lens to 200 μm below the inner surface of the ocellar lenses. The dorsal retina and ventral retina in each lens type are located at different distances from the back of the lens. In the description below we show images formed at BDs of 60 μm (ventral retina) and 100 μm (dorsal retina) for the median ocellus, 80 μm (ventral retina) and 140 μm (dorsal retina) for the lateral ocellus. These values approximately match the BFD measurements given above.

**Figure 5:**
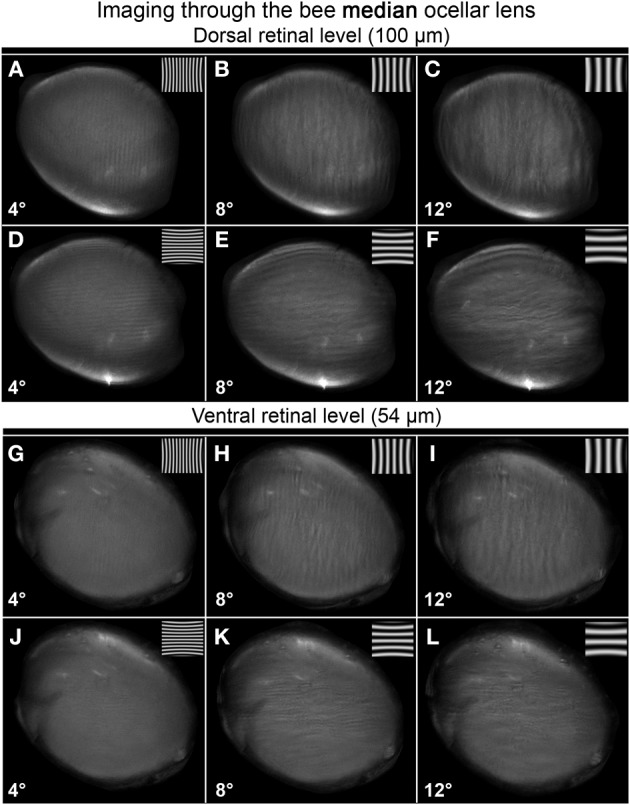
**Images of gratings through the honeybee median ocellus at the dorsal retinal level (A–F; 100 μm away from the back of the lens) and ventral retina (G–L; 54 μm away from the back of the lens)**. Spatial wavelength: ADGJ: 4°; BEHK: 8°; CFIL: 12°.

**Figure 6:**
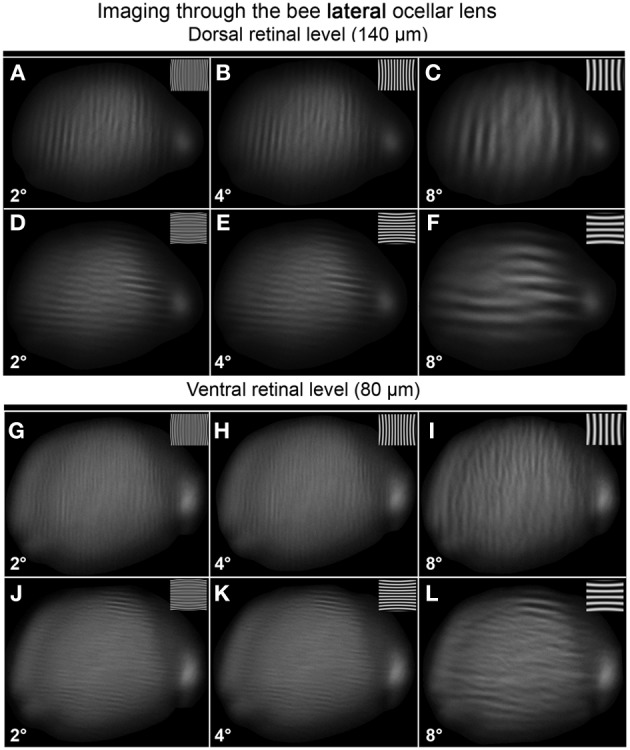
**Images of gratings through the honeybee lateral ocellus at the dorsal retinal level (A–F; 140 μm away from the back of the lens) and ventral retinal level (G–L; 80 μm away from the back of the lens)**. Spatial wavelength: ADGJ: 2°; BEHK: 4°; CFIL: 8°.

**Figure 7:**
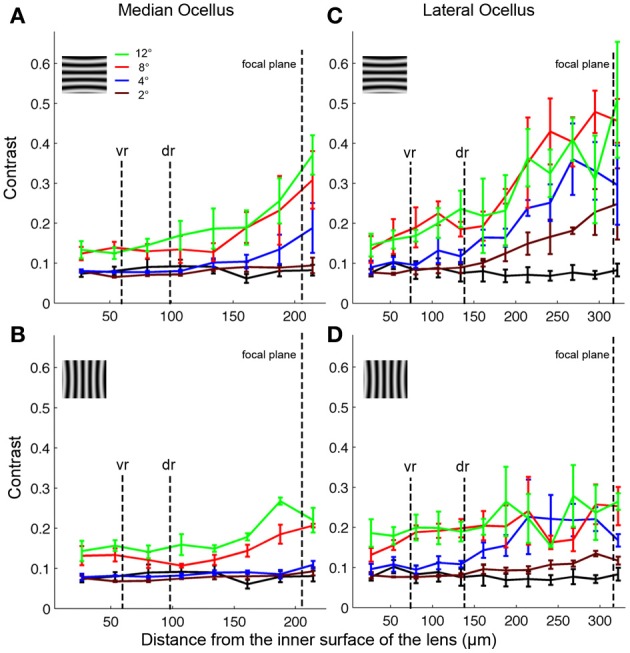
**Contrasts of images formed by the honeybee median (A,B) and lateral (C,D) ocellar lenses at different distances in relation to the inner surface of the tested lenses (mean ± SE, *n* = 3)**. The different colored lines show results from different spatial wavelengths. The black lines show the contrasts obtained for a blank screen. The focal plane, and the distances of the ventral (vr) and dorsal (dr) retinas are shown by vertical dashed lines. Inserts show the orientation of the gratings in each case (vertical or horizontal).

Both the median and lateral ocellar lenses were able to resolve spatial information at the dorsal and ventral retinal levels (Figures 5–7). This is evident from the fact that oriented gratings can be seen in most of the images shown in Figures 5, 6. However, based on qualitative visual inspection, there is a gradation of image quality. Gratings can be seen quite clearly when viewed through the dorsal retina of the median ocellus, at spatial wavelengths from 4 to 12° (Figures 5A–F). When images were viewed through the ventral retina of the median ocellus, gratings can be just discerned but they are of very poor quality (wavy and patchy) (Figures 5G–L). Images formed through the lateral ocelli are qualitatively better for both the ventral and dorsal retinas (Figures 6A–L). The orientation of the gratings can be clearly observed even with 2° patterns and the imaged gratings are quite sharp. By comparing the images at the ventral and dorsal levels of the lateral ocelli it is qualitatively evident that the images are sharper for the dorsal retina.

Armed with these impressions, we have quantified the results by considering the contrast in the collected images (Figure 7; mean ± SE, *n* = 3). We did this by measuring the maximum and minimum reflectance from the center of the images and then using those values to calculate the Michelson contrast (see Methods). Figure 7 plots the measured image contrast as functions of the distance from the inner surface of the lens (Back Distance, BD). We show this relationship for the median (left column) and lateral ocelli (right column), and for horizontal (top row) and vertical gratings (lower row).

The first step in the statistical analysis was to calculate the measured contrast using a blank input. We found that Michelson contrast was 0.07 with the blank stimulus, revealing that viewing a blank stimulus (with zero contrast) through the lenses generated a certain amount of noise (peaks and troughs in luminance). The measured contrast was significantly higher for all conditions at BDs close to the ventral and dorsal retinal levels for patterns with spatial periods of 8° or above (one-tailed *t*-test, *p* < 0.03). At lower spatial wavelengths, there was no significant difference in measured contrast between the blank images and the gratings except for BDs that were well beyond the retinal level. Nonetheless, inspection of Figures 5, 6 shows that the dark and light luminance regions for the low spatial wavelength patterns were more spatially ordered for the gratings compared to the blanks (i.e., alternating and oriented dark and light regions can be seen, rather than random patches of luminance). In conclusion, the contrast information for long spatial wavelength gratings is transferred through the ocellar lenses (median and lateral) better than low spatial wavelengths.

The second step was to compare the contrasts of the images through the median and lateral ocelli. For this we compared the measured contrast at the mid-point between the ventral and dorsal retinas for both ocellar types (i.e., midway between vr and dr, Figure 7). With one exception, for horizontal and vertical gratings with spatial periods of 2, 4, and 8° we found that the measured contrast was significantly higher for the lateral ocellus than for the median ocellus (one-tailed *t*-test, *p* < 0.05). The exception was for the 2° horizontal grating, which showed no significant difference in measured contrast between the median and lateral ocellus. When we used 12° patterns we found no significant difference in the contrast between images for the median and lateral ocelli. In conclusion, the lateral ocelli produce higher contrast images at the retinal levels than the median ocellus for both horizontal and vertical gratings for most spatial wavelengths tested. This confirms the qualitative impressions gained through visual inspection of the images in Figures 5, 6.

The third step was to compare images viewed at BDs that approximately equated to the ventral and dorsal retinas. There were no significant differences between the contrasts measured at the ventral or dorsal retinal levels for any of the tested spatial wavelengths, regardless of the orientation of the gratings. Therefore, there is no statistical evidence for improvements in spatial resolving power for the dorsal retinas compared to the ventral retinas in either the median or lateral ocelli.

### Anatomy of lateral ocellar descending neurons

**Figure 8:**
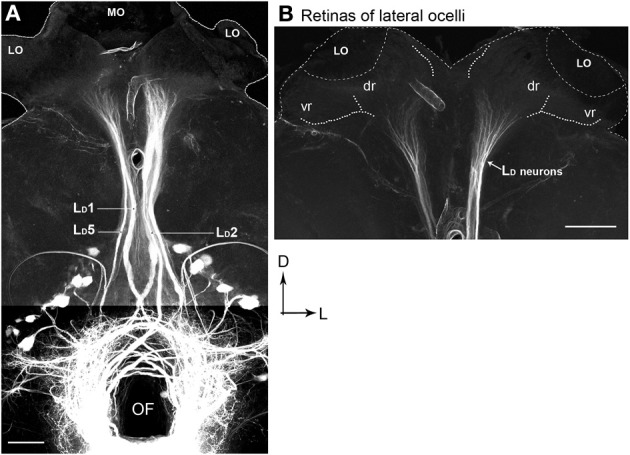
**Posterior views of stained descending neurons in the honeybee brain. (A)** All descending neurons filled when dye was placed in the dorsal region of the ventral nerve cord (including the medial dorsal track: MDT). **(B)** Many neurons are filled but only those that travel up into the ocelli are descending ocellar neurons (L_*D*_).

There are five pairs of lateral ocellar descending neurons in the honeybee (Pan and Goodman, 1977; Goodman, 1981; Milde and Homberg, 1984), referred to as L_*D*_1–L_*D*_5. These neurons have the unique feature that when dye is placed in the ventral nerve cord, they are the only neurons that send dendrites into the ocellar regions (Figure 8). We placed fluorescent dye into the dorsal tracts of the ventral nerve cord (Rehder, 1988), which filled all the L_*D*_ neurons (Figure 8A).

**Figure 9:**
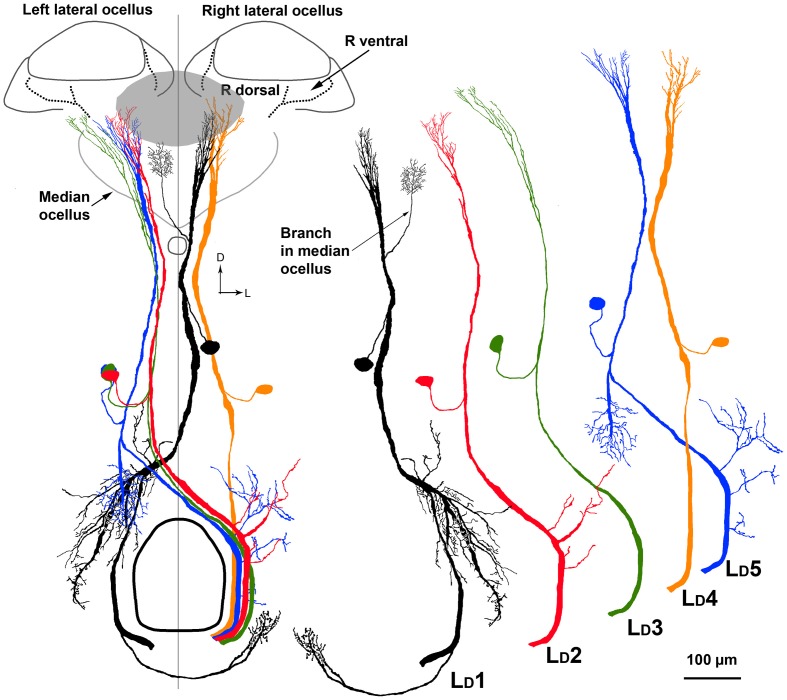
**On the left is a composite drawing showing the relationship between the five L_*D*_ neurons in relation to each other and to the ocellar retinas**. Outlined are the boundaries of the ocellar lenses and retinas. The gray patch shows the location of the dorsal retina of the median ocellus. On the right are individual drawings of the five cell types, to allow future identification. Scale bar = 100 μm.

The L_*D*_ neurons can be distinguished from each other by a series of clear features of their central brain morphologies. Given that when the ventral nerve cord is filled, only the descending ocellar neurons climb up into the ocellar tracts and that there are only five very large neurons on each side, it is easy to identify the main axons, cell bodies and the general locations of the dendrites with respect to the ocellar retinas (Figure 8A). It was more difficult to extract the dendritic structures of individual cells from the mass-fills but individual optical sections offered fine detail that allowed reasonable reconstruction over multiple sections (Figure 8B). Figure 9 shows the drawings of the five ocellar descending neurons. Some dendrites below the ocellar retinas in the mass fills proved to be impossible to assign unequivocally to a particular cell. The drawings present the dendrites that categorically belonged to the identified neurons. L_*D*_1 has a very distinctive branching pattern in the deutocerebrum, with a large branch that descends toward the suboesophageal ganglion. L_*D*_2 also has bushy dendrites in the deutocerebrum but without a single large branch. L_*D*_3 has a smaller axon and very fine dendrites in the ocellus. L_*D*_4 is the only neuron that descends into the same side of the ventral nerve cord as the ocellus that provides its input. L_*D*_5 is very characteristic because before it ascends the ocellar tract it swings very significantly to the contralateral side of the tracts.

Our primary interest was to determine which retinas within the ocelli provided input to the L_*D*_ neurons. Four L_*D*_ neurons (L_*D*_1, L_*D*_2, L_*D*_4, L_*D*_5) have their primary dendritic fields in the neural plexus that exits the dorsal retina of the lateral ocelli (Figures 8, 9). The vast majority of the dendrites from all four neurons overlap and occupy the central region of the dorsal retina of the lateral ocellus. Even though not every dendrite could be assigned with absolute certainty to a particular L_*D*_ neuron, it is clear that the dendrites from all four neurons are confined strictly to the dorsal retina of the lateral ocellus. L_*D*_3 does not send any branches into the dorsal retina of the lateral ocellus. However, it does send slender dendrites into the ventral retina of the lateral ocellus, close to the border between the ventral and dorsal retinas (Figure 8). L_*D*_1 was found to send fine bushy dendrites into the ventral retina of the median ocellus (Figure 9). The median ocellar branches from L_*D*_1 were close to the animal's midline but were biased toward the contralateral half of the retina (i.e., on the opposite side to the innervated lateral ocellus).

## Discussion

### The morphology of honeybee ocelli

The gross morphology of the honeybee ocelli has been investigated in two previous studies (Toh and Kuwabara, 1974; Ribi et al., 2011). Ribi et al. (2011) reported that honeybee ocelli have two distinct retinas. Here, for the first time we show 3-D models of the honeybee ocelli and their spatial resolving power. The model of the three ocelli is useful because it allows the lenses and retinas to be viewed from any perspective and provides a complete reconstruction of the functional structures. From the 3-D reconstructions of the ocelli we showed that the median ocellar lens is elongated downward and forms a complex non-spherical shape. The 3-D reconstructions can also be combined in the future with ray tracing (which involves calculating and modeling the light paths from different directions as it goes through the lenses) to give accurate mapping of the visual fields of the retinas.

Berry et al. (2011) found that the ocellar lenses of a nocturnal bee (*Megalopta genalis*) consist of at least three structurally distinct components, named the outer, middle and inner layer. The outer layer is stained lightly with Toluidine Blue; the middle layer is similarly composed of tightly layered tissue and stained more densely with Toluidine Blue; and the inner layer stains with the highest density of Toluidine Blue. The three-layered lens structure can also be observed in a crepuscular bee *Xylocopa tranquebarica* (Somanathan et al., 2009). In honeybees, the semi-thin sections through the ocelli showed that the honeybee ocellar lenses lack the middle layer and only possess the outer and inner layers. Although the Toluidine Blue indicates that there are different densities of organic material within the lens, it is still not known whether the different layers have different optical properties (Berry et al., 2011). It is possible that the non-homogeneous components of the ocellar lens may have different refractive indices, therefore helping to focus the images closer to the retinal plane (Berry et al., 2007b,c).

The dragonfly lateral ocellar lens has an asymmetrical inner surface, which reflects the underlying division of the lateral ocellar retina into two regions (Berry et al., 2007a,b). In honeybees, both the lateral and median ocellar lenses have been shown here to have an asymmetrical inner surface. The retina has two major regions, each with different lengths of rhabdomeres, which correspond with the shape of the lens. These two regions are referred to as the dorsal and ventral retinas. For the median ocellus, the retinal layer lies around the elongated side of the inner surface of the lens; for the lateral ocellus the retinal layer is on the inner surface of the lens that is close to the midline of the brain. In both cases the ocellar structure allows light from the horizon to travel further in the lens before it reaches the proximal limit of the dorsal retina, as compared to light from above, which lands on the ventral retina. Ribi et al. (2011) showed that the retinas form two distinctly separate nerve bundles as they pass to the first synaptic plexus where they make contact with second-order interneurons. Combining the 3-D structural reconstruction conducted here and the anatomical results suggest that the two retinal regions may have different roles in processing visual information. It is possible that the dorsal retina has evolved for receiving information about the horizon while the ventral retina has evolved to detect more general information regarding intensity changes in the sky above (Stavenga et al., 1979; Berry et al., 2007a,b,c; Ribi et al., 2011). The ventral retina may well be involved in the ocellar dorsal light response, as demonstrated in dragonflies (Stange and Howard, 1979) and locusts (van Kleef et al., 2013). The longer retinal cells in the dorsal retina may assist in capturing more light, which may be useful when viewing textured regions in front of the animals, close to the horizon. The shorter retinal cells in the ventral retina may not need to capture as much light because they usually face directly upwards, where the sky is brightest.

Previous studies have found that the structural morphology of Hymenopteran insects reflects their ecological niche. Two nocturnal species (halictid bee *Megalopta genalis* and paper wasp *Apoica pallens*) have a distal retinal surface that is positioned close to the proximal surface of the lens; while in the diurnal wasp (*Polistes occidentalis*) there is a gap between the retina and the proximal lens surface (Warrant et al., 2006). Frontal- and longitudinal-sections of honeybee ocelli reveal a similar structure as the diurnal wasp: both the median and lateral ocelli possess corneagal cells that contain screening pigments and a vitreous chamber that separates the inner surface of the lens and the retinal layer.

Although no direct measurements have been made of the visual field of the honeybee ocelli, it is possible to predict the field of vision based on the relative position of the ocelli, the hairless zone that surrounds the ocelli, the morphology of the lens and its relation to the retina. From the results here, it appears that the median ocellus looks up- and forward (covering the horizon), while the lateral ocelli look upwards and to either side of the head (also encompassing the horizon). More specifically, the 3-D modeling exercise has revealed that the ventral retinas of all ocelli take their primary input from the region directly above the head. In the most common body orientation, this would equate with the sky above the animal. However, when the animal's head rolls to one side the lateral ventral retina on one side would view the far darker horizon, possibly providing a strong clue to the animal's orientation (Stange and Howard, 1979). The dorsal retinas of all the honeybee ocelli view the horizon, suggesting that more sophisticated processing might occur through the dorsal retinas. This processing may well include the detection of motion (Goodman et al., 1990), as in dragonfly ocelli (van Kleef et al., 2008).

### Ocellar resolution

It has been consistently observed in a wide range of species that ocelli lack optical resolving power (Parry, 1947; Cornwell, 1955; Wilson, 1978; Schuppe and Hengstenberg, 1993; Warrant et al., 2006; Berry et al., 2007b,c). However, this view has recently been re-examined, with several studies showing that despite their poor focus, ocelli can still provide a certain level of spatial resolution (blowfly, *Calliphora erythrocephala*: Schuppe and Hengstenberg, 1993; diurnal and nocturnal wasps: Warrant, 2006; locusts, *Locusta migratoria*: Berry et al., 2007c; Dragonflies: van Kleef et al., 2005; Berry et al., 2006). From the back focal measurement results and the semi-thin sections of honeybee ocelli, we showed that both the honeybee median and lateral ocellar lenses form focal planes well beyond the proximal limit of the retina. However, a recent study on honeybee ocelli suggested that the focal plane of the honeybee median ocellus falls within the dorsal retina (Ribi et al., 2011). Comparing the back-focal-distances (BFDs) measured from the present study and that of Ribi et al., it is apparent that the BFDs for the lateral ocellar lenses are similar (both studies showed a BFD with a length of 320–332 μm), nevertheless, the BFDs for the median ocellar lens differed by approximately 50 μm (i.e., Ribi et al. reported it as 160 μm while our measurement was 209 μm). In the present study, the results were based the measurements from 10 animals (with 3 repeats in each animal), while in the study by Ribi et al., the results were from 3 repeats. The variation between the two studies may be due to the limitations of the hanging drop method. As the position of the tested lens on the saline drop is set by the surface tension of the drop and by gravity, it is not possible to finely control the angle between the lens and the viewing axis of the microscope. The other result that led to the conflicting conclusions is the measured distance between the lens and the retinas. Our direct measurements from histological sections show that the proximal limit of the dorsal retina from the back of the lens is 100 μm (see Figure 1), while the schematic diagram presented in Ribi et al. (2011) showed a distance of more than 200 μm. As a result of this, even if we account for the variation of the BFD measurements, the focal plane of the median ocellus is still beyond the proximal limit of the retinas. Therefore, we suggest that Ribi and colleagues made an error on the scale of the schematic diagram, which led to an incorrect conclusion. Based on our multiple observations, we conclude that the focal length of the honeybee median ocellus is beyond the proximal end of the median dorsal retina.

Despite the different distances from the back of the lenses to the proximal ends of the retinula cells in the ventral and dorsal retinas, we found no significant difference in the contrast of images as viewed by either retina. Therefore, at least based on this measurement, the longer retinula cells in the dorsal retinas do not offer a significant advantage in coding contrast. However, we did show that the lateral ocelli have significantly higher spatial resolution than the median ocellus. It is not immediately obvious why the lateral ocelli have higher resolving power.

### Descending neurons

Goodman (1981) provided a description of the L_*D*_ neurons in the bee. However, that analysis was done before the identification of two distinct retinas in the ocelli. Moreover, the exact position of the dendrites in the ocelli is not clear from that original work. Our goal was to establish exactly which retinas provided input to the identifiable cells, thus giving functional clues to their role in flight stability. We have shown that four of the five large descending neuron pairs restrict most of their dendrites to the dorsal retinas of the lateral ocelli (L_*D*_1, L_*D*_2, L_*D*_4, L_*D*_5). Interestingly, L_*D*_1 also sends fine dendrites into the border area between the ventral and dorsal retina of the median ocellus. It appears that most of the L_*D*_ neurons have evolved to extract information from the fronto-lateral horizon, via the dorsal retinas of the lateral ocelli. One of these neurons (L_*D*_1) also obtains information from the border region of the median ocellus. Among the 5 L_*D*_ neurons, only L_*D*_3 sends fine branches into the ventral retina of the lateral ocelli.

In dragonfly, it was shown that the L-neurons that innervate the dorsal retina of the lateral ocelli have a field of view at the horizon while the L-neuron that innervates the ventral retina is adapted for wide-field intensity summation (Berry et al., 2007a). Our 3-D simulation in Figure 4 shows that the information from the honeybee lateral ocelli would be useful for extracting information about movements of the head relative to the horizon during rolling movements. Parsons et al. (2006) demonstrated that in flies neurons appropriately combined information from the lateral ocelli and compound eyes to extract roll information. In honeybees, it is believed that the ocellar L-neurons play an important role in modulating motion-sensitive descending neuron activity (Guy et al., 1979; Goodman, 1981; Hung et al., 2013). Combined information from the lateral and median ocelli would also be useful for extracting information about dark/light transitions at the horizon associated with pitching body movements. It was previously noted that L_*D*_5 responds strongly to upward image motion in the frontal plane, simulating downward pitch of the body, but it is not known if this was a result of ocellar or compound eye input (Goodman et al., 1990). Other direction-selective, pitch sensitive descending neurons that do not have a direct input from the ocellar retinas have been shown to respond to ocellar stimulation (Hung et al., 2013).

Dendrites from the L_*D*_ neurons that innovate the median ocellar retina were reported in a few previous studies. Based on reconstructions of stained sections, Heinzeller (1976) drew a picture of the dendrites of a large descending ocellar neuron, which we can identify based on its brain anatomy as L_*D*_1. The resultant drawings imply that the dendrites of L_*D*_1 innervate the ventral retina of the median ocellus but not the lateral ocellus. This early finding is partially correct but the main branches in the lateral ocelli were clearly missed using the technique available at that time. Later, Goodman (1981) presented a summary drawing showing the brain and ocellar anatomies of all five L_*D*_ neurons. That drawing showed that all the neurons had their main dendritic branches in the lateral ocelli but also that two of the neurons, L_*D*_1 and L_*D*_3, sent minor dendritic branches into the median ocellus. Milde (1984; also see Milde and Homberg, 1984) stained 11 L_*D*_1 neurons in separate preparations, with only one having fine arborisations in the median ocellus. In that study, Milde also failed to find dendrites from L_*D*_3 extending into the median ocellus. He concluded that median branches from the L_*D*_ neurons were “the exception rather than the rule.” In our study we also found it difficult to identify dendrites in the median ocellar retina, however, using florescent confocal imaging techniques we were able to observe very fine median ocellar dendrites from L_*D*_1 in several preparations. However, we were not able to find dendrites from L_*D*_3 in the median ocellus.

An important point of difference between the results presented here and those presented by Goodman (1981) is that we show through optical scanning and neural tracing that the dendrites of four of the L_*D*_ neurons overlap significantly in the dorsal retinas of the lateral ocelli. Based on Goodman's drawings it appears that the dendrites are arranged in a clear retinotopic fashion, with each dendritic tree having little overlap. Moreover, it appears from those drawings that many of the dendrites occupy the ventral retina. It is probable that the drawings were based on single cell fills and that the summary picture presented shows superimposed single fills, thus not accurately capturing the exact placement of dendrites in the retinas and the degree of dendritic overlap. Our data show that the dendrites of the honeybee ocellar descending neurons largely overlap in the dorsal retina of the lateral ocellus. Dendritic overlap in L-neurons is also substantial in dragonfly ocelli (Berry et al., 2006), suggesting a degree of redundancy in which multiple cells view the same patch of visual space.

### Conflict of interest statement

The authors declare that the research was conducted in the absence of any commercial or financial relationships that could be construed as a potential conflict of interest.
